# Effects of warming on ectomycorrhizal colonization and nitrogen nutrition of *Picea asperata* seedlings grown in two contrasting forest ecosystems

**DOI:** 10.1038/srep17546

**Published:** 2015-12-10

**Authors:** Yuejiao Li, Didi Sun, Dandan Li, Zhenfeng Xu, Chunzhang Zhao, Honghui Lin, Qing Liu

**Affiliations:** 1 Key Laboratory of Mountain Ecological Restoration and Bioresource Utilization & Ecological Restoration Biodiversity Conservation Key Laboratory of Sichuan Province, Chengdu Institute of Biology, Chinese Academy of Sciences, P.O. Box 416, Chengdu 610041, P. R. China; 2 College of Life Sciences, Sichuan University, No.24 South Section 1, Yihuan Road, Chengdu 610065, P. R. China; 3 University of Chinese Academy of Sciences, No.19A Yuquan Road, Beijing 100049, P. R. China; 4 State Key Laboratory of Soil and Sustainable Agriculture, Institute of Soil Science, Chinese Academy of Sciences, Nanjing 210008, P. R. China; 5 Institute of Forest Ecology, Sichuan Agricultural University, Chengdu 611130, P. R. China

## Abstract

Ectomycorrhiza (ECM) plays an important role in plant nitrogen (N) nutrition and regulates plant responded to climate warming. We conducted a field experiment in a natural forest and a plantation in the eastern Tibetan Plateau to estimate the warming effects of open-top chambers (OTC) on ECM and N nutrition of *Picea asperata* seedlings. Four-year warming significantly decreased ECM colonization, ECM fungal biomass, fine root vigor, and the N concentration of leaf, stem and coarse root, but significantly increased fine root N concentration and N content of leaf, stem, fine root and whole plant in natural forest. Contrarily, warming induced no obvious change in most of these parameters in plantation. Moreover, warming decreased rhizospheric soil inorganic N content in both forests. Our results showed that four-year warming was not beneficial for ECM colonization of *P. asperata* seedlings in the two forests, and the seedlings in natural forest were more sensitive and flexible to experimental warming than in plantation. The changes of ECM colonization and fine root biomass for effective N uptake would be good for plant growth and remit N leaching under future warming in natural forest.

Ectomycorrhizal (ECM) fungi colonize roots, especially fine root tips to form symbioses with most temperate and boreal coniferous tree species^1^,^2^, acquiring carbohydrates (C) from the hosts, at the expense of providing their hosts with soil nitrogen (N) and other nutrients^3^. It is also well known that ECM colonization can increase seedling establishment and survival^4^, stimulate plant growth^5^ and enhance resistance to disease^6^ and abiotic stresses^3^. ECM root tips can be viewed as the main nutrient-absorbing organs, as much as 80% of total N content of some ECM trees originates from ECM symbionts^7^. ECM fungi facilitate nutrient uptake directly by increasing physical access to soil via extended extraradical hyphae, and changing root physiology^8^,^9^. In addition, ECM fungi was also reported to acquire N indirectly by increasing N availability of mycorrhizosphere or hyphosphere soil through release of enzymes^10^, and interaction with rhizosphere bacterial populations^11^. However, under extremely nutrient limited condition, negative effects of ECM colonization on the root nutrient uptake^12^, photosynthetic efficiency^13^ and host plant growth^14^ were also found. Whether host plant will profit from ECM colonization depended on the balances between the ECM fungi C cost and N supply for host plant^12^. When ECM failed to supply enough amounts of N, the host plant would decrease the C supply to ECM fungi and end their symbiotic relationship^15^.

Globally averaged surface temperature is predicted to increase by 1.4 °C to 5.8 °C over the period 1990 to 2100, and the temperature increase is more significant in higher altitudinal and latitudinal ecosystems^16^. Numerous studies addressing the effects of global warming on plant and ecosystem have focused on photosynthesis and aboveground plant growth^17^,^18^. With increasing recognition that roots play a key role in responses of plant and ecosystem to global warming^19^, more and more researchers pay attention to warming effects on root growth and root function related to plant nutrition especially plant N uptake and utilization in recent years^20^,^21^. Hence, the response of ECM symbiosis to warming gradually becomes one of the hot issue for their indispensable role in host root N acquisition and growth^22^,^23^. Global warming were reported to influence ECM symbiosis directly by changes of fine roots (physiology and mortality) and soil N availability^24^,^25^, and indirectly by above-ground changes of physiology and growth of their hosts^26^. Increased ECM colonization and abundance were commonly found with warmer temperature^27^,^28^,^29^. On the contrary, other studies also reported that experimental warming significantly decreased ECM colonization^2^,^30^. Thereby, the response of ECM to warming were different, as results of different environmental conditions, such as temperature, soil nutrient availability and moisture, etc^27^,^28^. In addition, plant might form symbiosis with microorganisms or enhance fine root biomass to improve fine root nutrient uptake efficiency under different environmental conditions^2^. Therefore, the warming effects on ECM colonization could also influence the plant nutrition. However, the changes in ECM colonization and the relationship between ECM and host N nutrition under future warming remained unclear and were still scarcely studied.

The subalpine coniferous forest ecosystems in the Eastern Tibetan Plateau are considered very sensitive to global climate warming^31^. Last century, large scale logging turned natural coniferous forests into clear cutting areas and dragon spruce (*Picea asperata Mast*.) were widely used for reforestation in such areas. Currently, there are over one million hectares of monoculture of *P. asperata* plantation in Western Sichuan. Our previous studies showed that deforestation and reforestation had induced great changes in soil biochemical properties, and further affected the initial responses of plants and forest soils to warming^32^,^33^. *P. asperata* as one of the key species is commonly associated with ECM fungi in this region. It was reported that experimental warming enhanced photosynthetic rates and biomass^34^, and changed plant nutrition by altering root growth and physiology of *P. asperata*^35^. However, these studies ignored the potential changes in ECM colonization, abundance, and the relationships of ECM and root physiology and N nutrition under warming. Besides, changes in land-use are also considered to affected ECM formation and biomass, and consequently regulate the responses of the host to climate warming^36^. Therefore, we conducted a field experiment in a natural forest and a dragon spruce plantation with OTC warming. On the basis of previous studies, we hypothesized that ECM colonization of *P. asperata* would increase and positively related with plant N content and root physiology under OTC warming condition, and the responses of ECM colonization and plant N nutrition to warming were different in the two forests.

## Results

### ECM colonization and ECM fungal biomass

**Figure 1:**
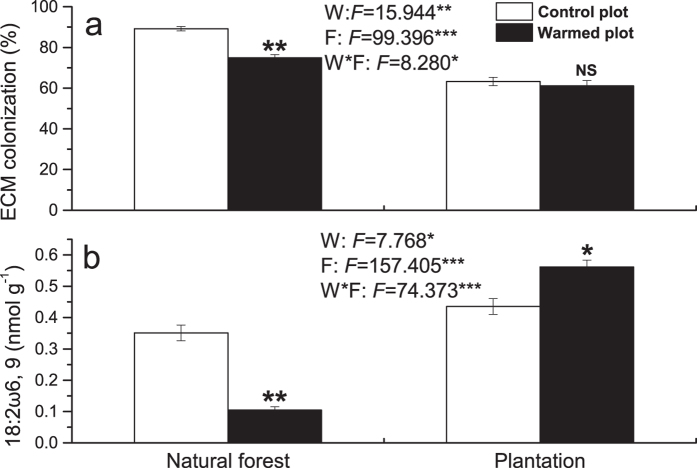
Effects of warming and forest type on ectomycorrhizal (ECM) colonization of *P. asperata* seedlings (a) and ECM fungal biomass in rhizospheric soil (b). Values indicate means ± SE, n = 3. Warming effects in both natural forest and plantation were assessed by student *t*-test, *F* and *P* values are given by two-way ANOVA for the effects of W: warming, F: forest type, and W*F: interaction of warming and forest type; *P < 0.05, **P < 0.01, ***P < 0.001, NS not significant.

Warming significantly decreased the ECM colonization and ECM fungal biomass by 16.1% and 70.2% in natural forest (Fig. 1). However, warming induced no significant effect on ECM colonization and increased ECM fungal biomass in plantation. In addition, ECM colonization and ECM fungal biomass was strongly influenced by forest type. Surprisingly, ECM colonization was remarkably higher, but ECM fungal biomass was less in natural forest compared with those in plantation.

### Rhizospheric soil inorganic N

**Figure 2:**
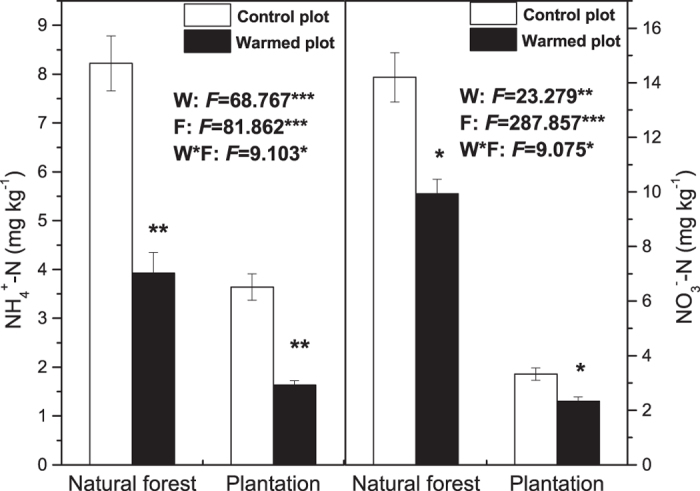
Effects of warming and forest type on the rhizospheric soil inorganic N (NH_4_^+^-N and NO_3_^−^-N). Values indicate means ± SE, n = 3. Warming effects in both natural forest and plantation were assessed by student *t*-test; *F* values are given by two-way ANOVA for the effects of W: warming, F: forest type, and W*F: interaction of warming and forest type; *P < 0.1; **P < 0.01; ***P < 0.001.

Soil NH_4_^+^-N and NO_3_^−^-N were significantly affected by warming and forest type, and there was significant interaction of warming and forest type. OTC warming significantly decreased natural forest and plantation soil NH_4_^+^-N by 52.2% and 55.8%, and NO_3_^−^-N by 30.1% and 29.5%, respectively. In addition, no whether warming or not, the soil NH_4_^+^-N and NO_3_^−^-N were higher in natural forest than those in plantation (Fig. 2).

### Plant biomass, N concentration and accumulation

Most components and total plant biomass of *P. asperata* seedlings were significantly affected by OTC warming, resulting in significant increase in natural forest (Table 1). Fine root and leaf biomass in natural forest were increased by 70.3% and 70.6%, respectively. However, only stem and total plant biomass were significantly increased by warming in plantation. In addition, whether warming or not, the ratio of root/shoot (R/S) were lower in natural forest than that in plantation.

**Figure 3:**
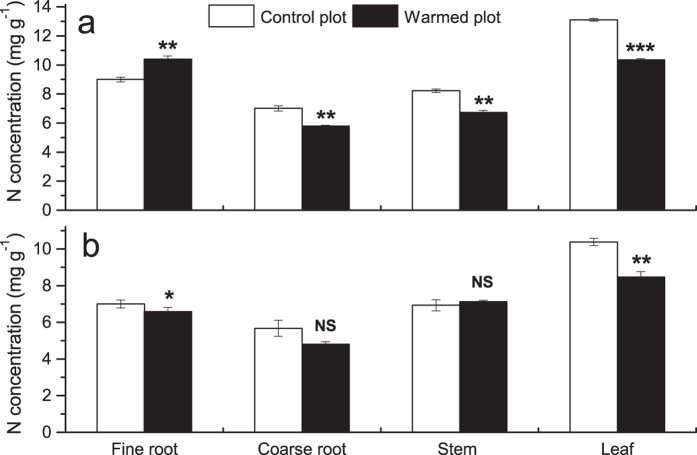
Effects of warming on nitrogen (N) concentrations of fine roots, coarse roots, stems and leaves of *P. asperata* seedlings grown in natural forest (a) and plantation (b). Values indicate means ± SE, n = 3. Warming effects in both natural forest and plantation were assessed by student *t*-test. *P < 0.05, **P < 0.01, ***P < 0.001, NS not significant.

The coarse root, stem and leaf N concentrations of seedlings grown in natural forest, and the leaf N concentrations of seedlings grown in plantation were significantly reduced by OTC warming (Fig. 3). In contrast, warming significantly increased the fine root N concentration by 15.5% in natural forest. However, no significant warming effects were found on root and stem N concentrations of seedlings grown in plantation.

Though N concentration of most components of the seedlings were significantly decreased, as a result of increased component biomass, the accumulated N contents in fine root, leaf, total plant of seedlings grown in natural forest were increased by warming about 95.8%, 34.7%, and 32.4%, respectively (Table 1). However, except stem N content, the component and total N contents showed no significant changes induced by warming in plantation. Furthermore, forest type had significant effects on all the plant tissue N concentrations (Table 2).

### Root vigor and NR activity

**Figure 4:**
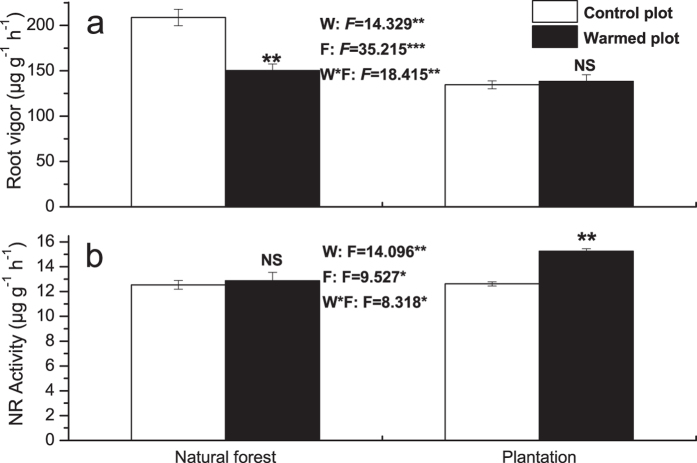
Effects of warming and forest type on root vigor (RV) (a) and nitrate reductase (NR) activity (b) of *P. asperata* seedlings. Values indicate means ± SE, n = 3. Warming effects in both natural forest and plantation were assessed by student *t*-test, *F* and *P* values are given by two-way ANOVA for the effects of W: warming, F: forest type, and W*F: interaction of warming and forest type; *P < 0.05, **P < 0.01, ***P < 0.001, NS not significant.

Root vigor and nitrate reductase (NR) activity were strongly influenced by warming and forest type. Significant interactive effects of warming and forest type were also found. Warming significantly decreased the root vigor by 28.1% in natural forest (Fig. 4a). Nevertheless, there was no significant effect of warming on root vigor in plantation. In contrast, no significant effect of warming was found on NR activity in natural forest, but there was a significant increase induced by warming in plantation (Fig. 4b).

### Relationships between ECM colonization and ECM fungal biomass, plant N concentrations, biomass, physiology and rhizosphere soil NH_4_^+^-N and NO_3_^−^-N content

ECM colonization was positively correlated with fine root N concentration (FN) (*r* = 0.704), coarse root N concentration (CN) (*r* = 0.785), stem N concentration (SN) (*r* = 0.631), leaf root N concentration (LN) (*r* = 0.904), and root vigor (*r* = 0.893) (Table 3). In addition, the soil NH_4_^+^-N (*r* = 0.883) and NO_3_^−^-N (*r* = 0.959) was also strongly positive correlated with ECM colonization. However, fine root biomass (FB) (*r* = −0.702), and coarse root biomass (CB) (*r* = −0.752) were negatively correlated with ECM colonization.

## Discussion

Contrary to our initial hypothesis, our results showed that warming was not beneficial for ECM colonization of seedlings in the two forests. The reduced ECM colonization were likely attributed to the shifts of soil microbial community structure induced by warming in natural forest^2^, since warming could change ECM community composition directly by warmer soil temperature^28^ and indirectly by decreasing rhizospheric soil inorganic N^37^. On the other hand, according to a recent study^29^, the increased growth of root by warming could enhance the proportion of non-mycorrhizal root, and thus decreased ECM colonization rate. In fact, ECM colonization was also found negatively correlated with fine root biomass in the present study. In addition, the reduction of ECM fungal biomass induced by warming was also responsible for the decreased ECM colonization in natural forest^38^. However, compared to the plantation, the lower ECM fungal biomass and the higher colonization rate were simultaneously observed in natural forest. Moreover, ECM fungal biomass was decreased in natural forest but increased in plantation as a result of warming. This disparity might because that ECM community structure was different in the two forests. Different ECM fungal types were diverse in their carbon costs to host plants and characteristics related to nutrient uptake such as hyphal morphology, cellular biochemistry and enzymatic capacity^3^. Thus, different ECM communities in the two forests were mainly contributed to the different responses of ECM fungal biomass and colonization to experimental warming^27^,^29^,^30^.

Ecosystem response could depend strongly on ecosystem initial conditions, such as initial turnover rates and stocks of soil organic matter, the plant and soil C pools, the dominant form of available N in the soil^39^. The responses of plant and soil to warming are likely to be complicated by land-use change^40^,^41^. Soil properties such as soil organic matter, inorganic N and total C, N contents were obviously affected by reforestation, and were much higher in natural forest than in plantation^32^. Similar with the responses of plant growth, soil organic matter and N mineralization of the two forests reported in our previous studies^32^,^42^, ECM colonization was more sensitive to warming in natural forest than that in plantation. Soil ECM fungal biomass in the two forest ecosystems were changed contrarily after four-year warming. This result indicated that the changes of soil organic matter, tree hosts and ECM community composition by deforestation and reforestation likely altered the main sources of C available for ECM, and might mostly contribute to the different responses of ECM fungal biomass and the symbiotic relationship between ECM fungi and host plant to experimental warming^43^. Furthermore, rhizospheric soil N availability could also alter ECM communities^37^ and influence ECM colonization. Thus, based on the different initial conditions of soil inorganic N in the two forests, ECM colonization and ECM fungal biomass responded differently to warming was reasonable.

N is the primary limiting element for plant productivity in most high-altitude and high-latitude ecosystems^39^. Experimental warming is reported to stimulate plant growth by enhancing photosynthesis, extending growing season, and increasing plant N uptake as a result of increasing soil N availability^18^,^44^. Our previous study also showed that one-year OTC warming significantly increased soil inorganic N due to increased net N mineralization rates, especially in natural forest^32^. However, the response of soil NH_4_^+^-N and NO_3_^−^-N varied with warming time. In the first year, warming induced high net N mineralization and availability might enhance plant N uptake and growth. The increased plant biomass especially below-ground biomass enhanced plant C supplied for soil microgram growth, and consequently increased microbial nitrogen immobilization in the subsequent year^44^. In addition, in the N-limited forests, the rate of plant N uptake was commonly higher than the rate of N converted to available forms in growth season^45^, however, in the non-growth season, soil N leaching possibly occurred when inorganic N accumulated in soil by enhanced N mineralization under warming condition^46^. Therefore, the decrease in the rhizospheric soil NH_4_^+^-N and NO_3_^−^-N contents after four-year OTC warming might attribute to increased plant N absorption, microbial nitrogen immobilization, and N leaching under chronic warming condition^45^,^46^.

In the present study, the decrease of tissue N concentrations induced by elevated temperature probably attributed to the alteration in N uptake, N allocation and growth or carbohydrate dilution^47^. However, leaf N concentration was significantly increased at the early stage of warming experiment^41^. The different responses of plant N concentration to warming time were reasonable because that soil inorganic N available for plant was reduced with warming time. This result suggested that although plant growth increased at the early stage, the benefits of warming to plant would be fade away as a result of soil N deficit in this region.

In agreement with previous studies, the closely positive correlations between ECM colonization and plant tissue N concentration indicated that ECM colonization might play an important role in plant N uptake^5^,^8^. Ectomycorrhizae could respond more directly and rapidly to climate change than their hosts^29^. The change of ECM colonization could affect plant N concentration directly by influencing the root physiology of the host. Root vigor, an important physiological parameter for evaluating nutrient uptake, was also positively correlated with N concentrations of most components, and varied consistently with the trend of ECM colonization among different treatments in this study. NR could stimulate the inorganic N assimilation in fine roots^48^,^49^, however, N concentration and ECM colonization were negatively correlated with NR activity. Therefore, ECM colonization might regulate root vigor to affect N uptake of *P. asperata* seedlings. However, as discussed above, the differences of plant N concentration between different forests and warming treatments might mostly attribute to the soil inorganic N content.

The increase in biomass larger than the decrease in tissue N concentration could be a reasonable explanation for the increased N contents of leaf, fine root and the whole plant in natural forest. However, there was no obvious change on the N content of coarse root, leaf and whole plant in plantation, though warming also significantly increased total biomass of the seedlings. These results suggested that the seedlings in natural forest had more advantages of N uptake under experimental warming condition. In the present study, ECM colonization and root vigor of *P. asperata* seedlings were significantly reduced in warmed plots of natural forest, but the biomass, N concentration and N content of fine root were significantly increased. It was suggested that the seedling might develop fine root to absorb more soil N when ECM colonization was decreased by warming. However, compared to natural forest, neither ECM colonization nor fine root biomass was changed in plantation. Additionally, there was no significant warming effects on root, leaf and whole plant N contents in plantation. *P. asperata* seedlings in natural forest were more sensitive and flexible to acclimate experimental warming.

In addition, N leaching is mainly responsible for the N lost in N-limited subalpine coniferous forests^32^. The N absorbing ability of plant could affect N leaching by changing soil inorganic N content^50^. In our study, the rhizospheric soil inorganic N significantly decreased by four-year warming in both forests, however, increased plant N accumulation was only observed in natural forest. Therefore, the enhanced N uptake of *P. asperata* seedlings might remit the N leaching and slow down the N lost in natural forest under global warming. And these findings further proved that N cycling processes in natural forest was stimulated by redistributing N between soil and plant pools^51^. On the other hand, the reduced soil inorganic N as a result of N leaching or soil microbial immobilization, might further aggravated N deficit for plant growth in plantation under long-term global warming.

In conclusion, the present study demonstrated that four-year experimental warming decreased ECM colonization and biomass, root vigor, and N concentration of most plant components, but increased the biomass and N concentration of the uptake organ (fine root) in natural forest, and consequently total N content of *P. asperata* seedlings were significantly increased. However, ECM colonization and plant N accumulation of the seedlings in plantation were insensitive to four-year OTC-warming. The different responses to warming in the two contrast forest would bring two disparate growth potential to the seedlings. In addition, the changes of ECM colonization and fine root biomass for effective N uptake was good for transferring soil N to plant N pool, and potentially remit the N leaching under future warming in natural forest ecosystem.

## Methods

### Field site and experimental design

The field sites were established at the Miyaluo Experimental forest of Lixian County, Eastern Tibetan Plateau (31°35′ N; 102°35′ E; 3,150 m a.s.l). The experiment was conducted in the 65-year-old dragon spruce plantation and the 200-year-old aspruce-fir-dominated natural forest. The plantation was approximately 300 m away from the natural forest. In late September 2008, six open top chambers (OTCs) were installed in each forest to simulate warming. Simutaneously, two-year-old *P. asperata* seedlings of uniform height and basal diameter were selected from a local nursery and transplanted in the center of each plot to avoid edge effects of OTC. Details of field site, experimental design, and basic soil properties of the two sites are described in previous studies^32^.

### Microclimate monitoring

In order to assess the OTC effects *in situ*, two automatic recording systems, one measure air temperature and air relatively humidity (RH) at 30 cm above the ground and another measure soil temperature at 5 cm depth, were set up in the center of three OTCs and three control plots, respectively. Data were taken at 60 min intervals during the experiment by alternating among sensors connected to a datalogger (Campbell AR5, Avalon, USA). 10 cm-depth soil moisture in the area of the OTC without rainfall interception was measured by hand-held probe (IMKO, Germany) once a week. OTC warming increased air and soil temperatures by approximately 1.32 °C and 0.66 °C in 2010, and averagely decreased soil moisture in natural forest and plantation by 3.49% and 4.43%, respectively, from April 2011 to April 2013. The detailed OTC warming effects on air temperature, soil temperature and moisture in natural forest and plantation were reported in previous study^32^,^52^.

### Plant and rhizospheric soil sampling

In early May 2013, 3 randomly chosen replicate seedlings in each plot were destructively harvested. Root systems adhering to a small amount of soil were separated from shoots by severing the plant at the root collar, and then the shoots were divided into leaf and stem components. The roots were shaken gently to separate soil not attach to the roots, and shaken vigorously by hand to collect the rhizospheric soil tightly adhered to roots. One composite rhizosphere soil sample per plot was collected by mixing 3 rhizospheric soil samples taken from 3 sampled plant roots. The composite soil samples were sieved (2 mm mesh size), and removed any visible plant material by hand. All the plant and soil samples were placed in plastic bags, labeled, transported on ice immediately. Plant samples were stored in 4 °C refrigerator until further processing. Each soil sample was divided into two subsamples. One was stored at 4 °C for inorganic N analysis, and the other was stored at −20 °C for the analysis of phospholipids fatty acid (PLFA) content.

### Root vigor and nitrate reductase activity assays

The root system of each seedling was soaked in distilled water and carefully rinsed clean of soil particles without disrupting the small root tips. The fresh intact lateral roots of each seedling were randomly chosen and blotted on absorbent paper for enzyme and root vigor assays.

Root vigor was measured based on the triphenylte-trazoliumchloride (TTC) method^53^. 0.3 g root were placed in tubes, filled with 5 ml of 0.4% TTC, 5 ml phosphate buffer (0.06 mol·l^−1^, pH 7.0). The tubes were incubated at 37 °C for 3 hours. The chemical reaction was stopped by adding 2 ml of 1 mol l-1 sulfuric acid in the tubes. This step was followed by extraction with triphenylformazan (TPF), which consisted of taking the root out of the tubes and put them in a mortar, added 3–4 ml of ethyl acetate and a little quartz sand and ground. The liquid phase was removed into a test tube. Added ethylacetate to the final volume 10 ml and recorded the absorbance at 485 nm. The absorbance values were used to calculate equivalent TPF concentrations with which the root activity was determined for each fresh root weight as follows:

root vigor (TPF ug^−1^FW hour^−1^) = TPF reduction (ug)/fresh weight (g)/time (h).

The nitrate reductase activity was assayed by an *in vitro* method modified according to Kaiser and Lewis^54^. 0.5 g prefrozen root was cut into 5 mm fragments, ground in a chilled mortar with quartz and pestle on ice, and then homogenized in 3 ml extraction buffer^48^. The homogenate was centrifuged at 4,000 × g for 15 min at 4 °C. 0.4 ml supernatant was mixed with 1.2 ml 0.1 M KNO_3_, 0.4 ml NADH (2 mg·ml^−l^) to a final volume of 2 ml. After incubation at 25 °C for 30 min, the reaction was terminated by the addition of 1 ml 1% (w/v) sulphanilamide and 1 ml 0.02% (w/v) N (l- napthyl) ethylenediamine dihydrochioride solution. Color developing for 15 min, the mixture was centrifuged at 4,000 × g for 5 min and then absorbance was recorded at 540 nm in a spectrophotometer.

### Determination of ECM colonization

8 randomly selected undamaged lateral roots per seedling were excised from the taproot. Then the lateral root were cut into approximately 1 cm fragments, put into a beaker containing distilled water, and thoroughly mixed. 30 root fragments per seedling were randomly selected and placed in a Petri dish to determine the mycorrhizal colonization of first order roots using a stereomicroscope (Stemi SV 11; Zeiss, Jena, Germany). The first order roots were classified as vital or non-vital root tips. Vital root tips were identified as ectomycorrhizal or non-ectomycorrhizal depends on the presence or absence of ectomycorrhizal mental^55^. Non-vital root tips with a shrunken appearance and an easily detachable cortex were excluded from observations^56^. Ectomycorrhizal colonization (%) of first order roots (per root fragment or seedling) was calculated as: Ectomycorrhizal Colonization (%) = Ectomycorrhizal root tips × 100/(Ectomycorrhizal root tips + Vital non-mycorrhizal root tips)^56^.

### Analyses of plant biomass, N concentrations and N contents

The remaining roots were divided into fine roots (<2 mm) and coarse roots (2 mm) according to the root diameter, then samples of leaf, stem, fine root and coarse roots were dried in an oven at 65 °C for 48 h and the dry mass of each plant tissue were weighed. Then, the dry samples were ground with a ball mill to a fine powder (SPEX 8000D, Edison, America). Powdered dry samples were weighed into tin cartridges (Hekatech, Wegberg, Germany) and analyzed for total N using an element analyzer (Vario MACRO, Elementar Analysesyteme GmbH, Hanua, Germany). And the N content was calculated with plant tissue N concentrations (mg·g^−1^) and biomass (g) as follows:

Tissue N content(mg) = tissue N concentration × tissue biomass


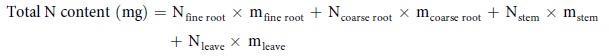


N_fine root_, N_coarse root_, N_stem_, and N_leave_ are the N concentration of fine roost, coarse roots, stems, and leaves. And m_fine root_, m_coarse root_, m_stem_, and m_leave_ are the biomass of fine roost, coarse roots, stems, and leaves.

### Analyses of rhizospheric soil ECM fungal biomass and soil inorganic N (NH_4_^+^-N and NO_3_^−^-N)

PLFA analysis was performed with the procedure according to Bossio and Scow^57^. Triplicate subsamples of fresh rhizospheric soil equivalent to 8 g dry soil were extracted, fractionated, and methyl esterified. The fatty-acid methyl esters were extracted with n-hexane and dried under N_2_. The dried samples were redissolved in hexane containing the fatty acid 19:0 as an internal standard. Then identification of the fatty acid methyl esters was performed using the standard EUKARY chromatographic program (MIDI, Microbial ID, Inc., Newark, DE, USA) based on chromatographic retention time. The relative content of individual fatty acids was calculated according to the peak area and internal standard curve, and expressed as mole percentage (nmol·g^−1^ soil). The PLFA 18:2ω6,9 was used as an indicator of ECM biomass^38^,^58^.

Rhizospheric soil inorganic N (NH_4_^+^-N and NO_3_^−^-N) was extracted from sieved soil samples with 2 M KCl and measured by colorimetry.

### Statistical analyses

All analyses were performed using SPSS 17.0. Before analysis, all data were tested for the homoscedasticity. If data were heterogeneous, they were ln-transformed before analysis. A two-way analysis of variance was used to test the effects of warming, forest type and their interactions on all of the variables. For specific forest type, Student *t*-tests were used to compare the effect of the experimental warming. We also used Pearson’s correlation analyses to examine the relationships between ECM colonization, ECM fungal biomass, plant tissue N concentrations, plant tissue biomass, root physiology parameters (RV and NR activity) and rhizosphere soil inorganic N (NH_4_^+^-N and NO_3_^−^-N). The statistical tests were considered significant at the *P* < 0.05 level.

## Additional Information

**How to cite this article**: Li, Y. *et al*. Effects of warming on ectomycorrhizal colonization and nitrogen nutrition of *Picea asperata* seedlings grown in two contrasting forest ecosystems. *Sci. Rep.* **5**, 17546; doi: 10.1038/srep17546 (2015).

## Figures and Tables

**Table 1:** Effects of warming and forest type on plant biomass (FB, CB, SB, LB, and TB), the ratio of root/shoot (R/S), and plant Nitrogen contents (FNC, CNC, SNC, LNC, and TNC) of *P. asperata.*

	Natural forest			Plantation			P-value		
	Unwarming	Warming	t-test	Unwarming	Warming	t-test	W	F	W*F
FB (g)	1.01 ± 0.09	1.72 ± 0.13	**0.010**	1.57 ± 0.26	2.25 ± 0.20	0.107	**0.005**	**0.017**	0.936
CB (g)	1.50 ± 0.10	1.79 ± 0.17	0.203	2.05 ± 0.18	2.58 ± 0.18	0.100	**0.031**	**0.003**	0.462
SB (g)	6.50 ± 0.34	9.97 ± 0.50	**0.005**	6.73 ± 0.56	8.96 ± 0.21	**0.021**	**<0.001**	0.391	0.189
LB (g)	5.10 ± 0.17	8.70 ± 0.25	** < 0.001**	5.68 ± 0.37	6.69 ± 0.23	0.082	**<0.001**	**0.027**	**0.001**
TB (g)	14.11 ± 0.63	22.17 ± 0.84	**0.002**	16.03 ± 1.36	20.48 ± 0.81	**0.048**	**<0.001**	0.909	0.094
R/S	0.216 ± 0.012	0.188 ± 0.014	0.206	0.289 ± 0.014	0.308 ± 0.015	0.436	0.731	**<0.001**	0.138
FNC (mg)	9.11 ± 0.89	17.84 ± 1.39	**0.006**	11.03 ± 1.98	14.69 ± 0.87	0.165	**0.002**	0.661	0.099
CNC (mg)	10.48 ± 0.42	10.36 ± 0.92	0.909	11.46 ± 0.30	12.41 ± 1.05	0.432	0.594	0.076	0.492
SNC (mg)	53.55 ± 3.45	67.08 ± 3.75	0.057	46.30 ± 2.22	63.81 ± 2.01	**0.004**	**0.001**	0.113	0.520
LNC (mg)	66.85 ± 2.10	90.04 ± 2.57	**0.002**	59.14 ± 4.91	56.50 ± 0.25	0.620	**0.009**	**<0.001**	**0.002**
TNC (mg)	139.99 ± 6.24	185.32 ± 7.70	**0.010**	127.93 ± 8.87	147.41 ± 4.01	0.116	**0.002**	**0.007**	0.100

Values indicate means ± SE, n = 3. FB, fine root biomass; CB coarse root biomass; SB, stem biomass; LB, leaf biomass; TB, total plant biomass; FNC, fine root nitrogen content; CNC, coarse root nitrogen content; SNC, stem nitrogen content; LNC, leaf nitrogen content; TNC, total plant nitrogen content. Warming effects in both natural forest and plantation were assessed by student *t*-test; *F* values are given by two-way ANOVA for the effects of W: warming, F: forest type, and W*F: interaction of warming and forest type. *P* < 0.05 are bold.

**Table 2:** Results of two-way ANOVA showing the P values for responses of fine root, coarse root, stem, and leaf N concentrations of *P. asperata* to warming and forest type.

Factor	N concentrations (mg·g^−1^)			
	Fine root	Coarse root	Stem	Leaf
W	0.053	**0.003**	**0.006**	**<0.001**
F	**<0.001**	**0.001**	**0.033**	**<0.001**
W*F	**0.003**	0.492	**0.001**	0.054

W: warming, F: forest type, and W*F: interaction of warming and forest type. *P* < 0.05 are bold.

**Table 3:** Pearson’s correlation coefficients between ECM colonization (ECMc) and plant N concentrations (FN, CN, SN, and LN), biomass (FB, CB, SB, LB, and TB), physiology (RV and NR) and rhizosphere soil NH_4_^+^-N and NO_3_^−^-N content across all of the treatments.

	ECMB	FN	CN	SN	LN	FB	CB	SB	LB	TB	RV	NR	NH_4_^ + ^-N	NO_3_^−^-N
ECMc	−0.500	0.704*	0.785**	0.631*	0.904***	−0.702*	−0.752**	−0.223	−0.165	−0.366	0.893***	−0.538	0.883***	0.959***
ECMB	1	−0.947***	−0.355	0.144	−0.367	0.311	0.592*	−0.368	−0.596*	−0.285	−0.199	0.535	−0.292	−0.603*
FN		1	0.489	0.093	0.511	−0.402	−0.646*	0.331	0.502	0.210	0.406	−0.537	0.458	0.789**
CN			1	0.703*	0.880***	−0.890***	−0.860***	−0.589*	−0.436	−0.682*	0.733**	−0.606*	0.916***	0.812**
SN				1	0.687*	−0.557	−0.390	−0.575	−0.638*	−0.648*	0.746**	−0.212	0.722**	0.597*
LN					1	−0.849***	−0.815**	−0.564	−0.452	−0.665*	0.860***	−0.688*	0.960***	0.858***
FB						1	0.913***	0.664*	0.520	0.772**	−0.671*	0.611*	−0.833**	−0.683*
CB							1	0.427	0.223	0.544	−0.657*	0.582*	−0.810**	−0.777**
SB								1	0.940***	0.981***	−0.449	0.255	−0.587*	−0.138
LB									1	0.931***	−0.443	0.061	−0.480	−0.039
TB										1	−0.563	0.303	−0.683*	−0.277
RV											1	−0.261	0.899***	0.814***
NR												1	−0.568	−0.589*
NH_4_^ + ^-N													1	0.870***
NO_3_^−^-N														1

ECMc, ectomycorrhizal colonization; ECMB, ectomycorrhizal fungal biomass; FN, fine root N concentration; CN, coarse root N concentration; SN, stem N concentration; LN, leaf N concentration; FB, fine root biomass; CB, coarse root biomass; SB, stem biomass; LB, leaf biomass; TB, total plant biomass; RV, root vigor; NR, nitrate reductase activity;. *P < 0.05, **P < 0.01, ***P < 0.001.
